# CO_2_ Electroreduction in Ionic Liquids

**DOI:** 10.3389/fchem.2019.00102

**Published:** 2019-03-04

**Authors:** Deonildo Faggion, Wellington D. G. Gonçalves, Jairton Dupont

**Affiliations:** Laboratory of Molecular Catalysis, Institute of Chemistry, Universidade Federal do Rio Grande do Sul, Porto Alegre, Brazil

**Keywords:** ionic liquids, carbon dioxide, electrochemistry, reduction, oxidation

## Abstract

CO_2_ electroreduction is among the most promising approaches used to transform this green-house gas into useful fuels and chemicals. Ionic liquids (ILs) have already proved to be the adequate media for CO_2_ dissolution, activation, and stabilization of radical and ionic electrochemical active species in aqueous solutions. In general, IL electrolytes reduce the overpotential, increase the current density, and allow for the modulation of solution pH, driving product selectivity. However, little is known about the main role of these salts in the CO_2_ reduction process the assumption that ILs form solvent-separated ions. However, most of the ILs in solution are better described as anisotropic fluids and display properties of an extended cooperative network of supramolecular species. That strongly reflects their mesoscopic and nanoscopic organization, inducing different processes in CO_2_ reduction compared to those observed in classical electrolyte solutions. The major aspects concerning the relationship between the structural organization of ILs and the electrochemical reduction of CO_2_ will be critically discussed considering selected recent examples.

## Introduction

The reduction of atmospheric carbon dioxide (CO_2_) is one of the major challenges of modern life. This is due to the atmospheric increase in this gas by contemporary industrial activity and its contribution to possible global warming issues, the consequences of which can affect the future generation (Mac Dowell et al., 2017). Hence, alternative sources of energy that decrease the use of fossil fuels, as well as the reduction of the CO_2_ concentration in the air atmosphere, are required. One of the most elegant ways to achieve this objective is the catalytic transformation of CO_2_ into C1 feedstocks and fuels.

Efforts have been undertaken to use the sustainable energy of sunlight, directly or indirectly, to convert CO_2_ by photocatalytic chemistry (Sasirekha et al., 2006; Habisreutinger et al., 2013; Dong et al., 2018; Lin et al., 2018), electrochemical (Dong et al., 2018; Francke et al., 2018; Resasco et al., 2018; Yuan et al., 2018), and photo-electrochemical approaches (Barton et al., 2008; Kaneco et al., 2009; Sahara et al., 2016). For a sustainable and high energy efficiency process, CO_2_ electrochemical reduction reaction (CO_2_ERR) is expected to exhibit a high Faradaic efficiency at a low overpotential. In this vein, ionic liquids (ILs) are among the most promising materials under investigation due their unique physico-chemical properties.

This is mainly due to IL selectivity and relatively high CO_2_ absorption capacity, as well as their ability to stabilize charged CO_2_ species (Shkrob and Wishart, 2009). ILs also present a wide electrochemical window (Hayyan et al., 2013), thermal and chemical stability (Cao and Mu, 2014), negligible volatility (Anthony et al., 2001), and possible use as electron transfer mediators for redox catalysis (Balasubramanian et al., 2006), which makes them an interesting alternative to promote the CO_2_ERR. The technology for using CO_2_ as a renewable energy carrier is still far from practical application, making the design of novel electrochemistry technologies using ILs for the CO_2_ERR a “hot” field for recent research.

The real challenge for sustainable and high energy efficiency processes, and turning them into practical alternatives, is to develop a way to lower the energy barrier for CO_2_ERR due to the high stability of this compound. Decreasing the overvoltage of the reaction as much as possible will make the CO_2_ fixation costs low enough for practical use (Haran et al., 1998).

The objective of the present review is to highlight the use of ILs for CO_2_ERR, and the influence in the reactions that have been attempted to this purpose. CO_2_ERR using ILs is able to provide high product selectivity and conversion efficiency (Alvarez-Guerra et al., 2015) (Table 1). There are several reviews on electrochemistry in ILs, but the main aspects related to the roles of these fluids are only marginally treated for specific applications (Buzzeo et al., 2004; Silvester and Compton, 2006; Hapiot and Lagrost, 2008; Ohno and Fukumoto, 2008; Rees and Compton, 2011).

**Table 1:** Selected examples of CO_2_ERR employing ionic liquids.

**Entry**	**Electrode**	**Onset potential (V)**	**Electrolysis potential (V)**	**Faradaic efficiency product (%)**	**Current density (mA cm^**−2**^)**	**Electrolyte**	**References**
1	Ag	reductive peak −1.61 V vs. Ag/AgI	−1.80 V vs. Ag/AgI	dimethyl carbonate (74)	charge passed, 1.0 F.mol^−1^	Bare [BMIM][BF_4_]	Zhang et al., 2008
2	Pt disk	reductive peak −1.8 V vs. silver wire	−1.8 V vs. silver wire	n/a	5.7	[EMIM][BF_3_Cl]	Snuffin et al., 2011
3	Au	n/a	−1.16 vs. Ag/AgCl	CO (85)	7	0.1 mol dm^−3^ KHCO_3_	Ohmori et al., 2001
4	Ag	n/a	−1.50 vs. cell potencial	CO (96)	n/a	18% [EMIM][BF_4_] in water	Rosen et al., 2011
5	Bi-CMEC	−1.80 vs. SCE	−2.00 vs. SCE	CO (82)	31	[EMIM][PF_6_]	Medina-Ramos et al., 2014
6	Bi-CMEC	−1.80 vs. SCE	−2.00 vs. SCE	CO (82)	26	[EMIM][BF_4_]	Medina-Ramos et al., 2014
7	Bi-CMEC	−1.80 vs. SCE	−2.00 vs. SCE	CO (79)	17	[BMIM][Cl]	Medina-Ramos et al., 2014
8	Bi-CMEC	−1.80 vs. SCE	−2.00 vs. SCE	CO (74)	20	[BMIM][Br]	Medina-Ramos et al., 2014
9	Bi-CMEC	−1.80 vs. SCE	−2.00 vs. SCE	CO (87)	25	[BMIM][OTf]	Medina-Ramos et al., 2014
10	Imidazole incorporated into a phosphonium-type IL-modified Au electrode	−0.32 vs. Ag/AgCl	−0.80 vs. Ag/AgCl	CH_3_OH (9) HCOOH (30) CO (5)	0.095	0.1 mol dm^−3^ NaClO_4_	Iijima et al., 2018
11	Pb	−2.30 vs. Ag/AgNO_3_	−2.40 vs. Ag/AgNO_3_	Oxalate (78) CO (10)	0.6	0.1 mol dm^−3^ TEAP/ACN	Sun et al., 2014
12	Pb	−2.12 vs. Ag/AgNO_3_	−2.25 vs. Ag/AgNO_3_	Carboxylate (55) CO (42)	0.6	0.1 mol dm^−3^ [EMIM][NTf_2_]/ACN	Sun et al., 2014
13	MoO_2_/Pb	−2.22 vs. Fc/Fc+	−2.45 vs. Fc/Fc+	${\text{HCO}}_{2}^{-}$ (38) C_2_${\text{O}}_{4}^{2-}$ (6) CO (41)	20	0.3 M [BMIM][PF_6_] in ACN	Oh and Hu, 2015
14	MoO_2_/Pb	−2.22 vs. Fc/Fc+	−2.45 vs. Fc/Fc+	${\text{HCO}}_{2}^{-}$ (18) C_2_${\text{O}}_{4}^{2-}$ (5) CO (60) H_2_ (12)	n/a	0.3 mol dm^−3^ [BMIM][PF_6_] in ACN + 0.1 mol dm^−3^ water	Oh and Hu, 2015
15	MoO_2_/Pb	−2.22 vs. Fc/Fc+	−2.45 vs. Fc/Fc+	${\text{HCO}}_{2}^{-}$ (10) C_2_${\text{O}}_{4}^{2-}$ (5) CO (52) H_2_ (25)	n/a	0.3 mol dm^−3^ [BMIM][PF_6_] in ACN + 0.2 mol dm^−3^ water	Oh and Hu, 2015
15	MoO_2_/Pb	−2.22 vs. Fc/Fc+	−2.45 vs. Fc/Fc+	HCO_2−_ (6) C_2_${\text{O}}_{4}^{2-}$ (4) CO (51) H_2_ (29)	n/a	0.3 mol dm^−3^ [BMIM][PF_6_] in ACN + 0.3 mol dm^−3^ water	Oh and Hu, 2015
16	Ag	~−0.62 vs. Ag/AgNO_3_	−0.70 vs. Ag/AgNO_3_	${\text{HCO}}_{2}^{-}$ (95)	Charge (10 C)	0.1 mol dm^−3^ [P_66614_][124Triz] in ACN + 0.7 mol dm^−3^ of water	Hollingsworth et al., 2015
17	Ag	~−0.62 vs. Ag/AgNO_3_	−1.90 vs. Ag/AgNO_3_	${\text{HCO}}_{2}^{-}$ (6) CO (6) H_2_ (41)	Charge (10 C)	0.1 mol dm^−3^ [P_66614_][124Triz] in ACN + 0.7 mol dm^−3^ of water	Hollingsworth et al., 2015
18	Ag	reductive peak −1.5 V vs. Cc+ /Cc	n/a	n/a	0.7 mA.cm^−2^	Bare [PMIM][NTf_2_]	Tanner et al., 2016
19	Ag	reductive peak −1.5 V vs. Cc+ /Cc	n/a	n/a	−1.5	Bare [EMIM][NTf_2_]	Tanner et al., 2016
20	Ag	reductive peak (−1.1 V vs. Cc^+^/Cc)	n/a	n/a	−1.60	Bare [BMIM][NTf_2_]	Tanner et al., 2016
21	Ag	reductive peak −1.05 V vs. Cc+ /Cc	n/a	n/a	−1.5	Bare[BMIM][NTf_2_]	Tanner et al., 2016
22	Ag	reductive peak −1.05 V vs. Cc+ /Cc	n/a	n/a	−0.8	Bare [BMIM][BF_4_]	Tanner et al., 2016
23	Ag	reductive peak −1.6 V vs. Cc+ /Cc	n/a	n/a	0.75	Bare [BMIM][FAP]	Tanner et al., 2016
24	Ag	−2.20 V vs. Fc+/Fc	−2.4 V vs. Fc+/Fc	n/a	~10.0	0.1 M [Bu_4_N][PF_6_] + 0.02 M [Ethyl 2-Methyl Imimidazolium][BF_4_] +	Lau et al., 2016
25	Ag	−2.15 V vs. Fc+/Fc	−2.4 V vs. Fc+/Fc	n/a	~16.0	0.1 M [Bu_4_N][PF_6_] + 0.02 M [Ethyl 2,3- dimethyl Imimidazolium][BF_4_]	Lau et al., 2016
26	Ag	−2.30 V vs. Fc+/Fc	−2.4 V vs. Fc+/Fc	n/a	~5.5	0.1 M [Bu_4_N][PF_6_] + 0.02 M [Ethyl 2,3,4,5- tetramethyl Imimidazolium][BF_4_][BF_4_]	Lau et al., 2016

The role of the IL has been described as mainly absorbing CO_2_ and stabilizing the ${\text{CO}}_{2}^{•-}$ (radical anion) that is related to the electronic properties imposed by both the cation and anion. It appears that in ILs containing basic anions the role of the IL is not only related to the formation and stabilization of ${\text{CO}}_{2}^{•-}$, but also the pH control of the reaction mixture. We will first briefly discuss the structural organization of bare ILs and solutions of ILs. Second, the formation and stabilization of ${\text{CO}}_{2}^{-}$ in solutions (aqueous and organic) of ILs associated with non-basic anions will be addressed. Thirdly, CO_2_ERR employing ILs containing basic anions, in which the role of bicarbonate and buffering will be detailed. Finally, the influence of the macroscopic and nanoscopic properties of ILs in solution on CO_2_ diffusion and electrochemical activation are discussed considering the most recently published results.

## Bare ILs and IL Solutions

The well-known and unique physical-chemical properties cited above are attributed to the structural organization of bare ILs, which are highly ordered fluids described as a well-organized hydrogen-bonded polymeric supramolecular structure in the solid, liquid phase and is apparently maintained to a great extent even in the gas phase. The most investigated classes of ILs are imidazolium salts, and their properties can be finely tuned by varying the N-alkylimidazolium substituents (Dupont, 2004).

However, taking into account that water-free ILs are extremely difficult to obtain, it is expected that even traces of water may present a profound effect on the organization and reactivity of ILs at the nanoscopic level. Hence, it is important to consider the presence of water when employing and analyzing physico-chemical IL properties (Zanatta et al., 2016).

In the case of an aqueous system, the values of standard reduction potentials (SRP) can be influenced by the water and proton concentration. This effect can be derived from the activity coefficients of the water and protons in solution. The consequence of 18 mol% water in 1-butyl-3-methylimidazolium tetrafluoroborate ([BMIM][BF_4_]) is a 6 mV shift of the SRP for the bare IL, and the addition of 0.1 M HCl shifts the SRP by 28 mV (Kim et al., 2004; Matsubara et al., 2015). This effect causes an imprecise determination of the real SRP in CO_2_ERR, resulting in lower overpotentials in comparison to the real decreasing overpotential, making a precise comparison impossible (Matsubara et al., 2015).

When other molecules are introduced into this organization, a disruption of the hydrogen bond network occurs, generating nanostructures with polar and non-polar regions. Under this condition, the concept of polarity of the solvent, generally used to describe other solvents, cannot be applied to ILs (Dupont, 2004). This collapsed macrostructure starts to form contact ion pair structures, and in an infinite diluted solution can form a solvent-separated ion pair network (Stassen et al., 2015).

There is a general misunderstanding when correlating the physical-chemical properties attributed to bare ILs when the studies are made in a different concentration regime, i.e., with the addition of other species or solvents in the media (MacFarlane et al., 2017).

## ${\text{CO}}_{2}^{•-}$ Radical in ILs

After the confirmation in 2007 that ILs are able to boost organic carbonate synthesis by electrochemistry under ambient conditions (Zhang et al., 2008) (Table 1, entry 1), CO_2_ERR with ILs has grown exponentially due to the kinetic effects that minimize the energy necessary for intermediate ${\text{CO}}_{2}^{•-}$ formation.

The 1-ethyl-3-methyl-imidazolium trifluorochloroborate ([EMIM][BF_3_Cl]) IL can bind to CO_2_ through a Lewis base adduct, becoming active for CO_2_ERR and showing a high faradaic efficiency at low overpotentials (Snuffin et al., 2011) (Table 1, entry 2). The capability of lowering the overpotential for CO_2_ERR was also confirmed when using [EMIM][BF_4_] to “stabilize” ${\text{CO}}_{2}^{•-}$ (Rosen et al., 2011).

The studies presented in this review have shown that ILs are among the most efficient materials as both electrolytes and active functionalized materials for CO_2_ERR. Therefore, they may constitute a key compound in the development of new technologies for large-scale applicability. The most recent report showed that methylimidazolium groups can be attached to the periphery of an iron porphyrin, providing a pre-organized environment that presents excellent selectivity for CO production at low overpotentials, with water as a solvent and proton source (Khadhraoui et al., 2018).

However, until now, the precise mechanisms by which ILs decrease the overpotential have not been completely elucidated. In many cases, even the global electrochemical reactions were not clarified, making it difficult to determine the SRP and the real decrease in the reaction overpotential. It can be demonstrated by the simple modification of the imidazolium cation, able to act like a proton source to the CO_2_RR (Matsubara et al., 2015), and changes the equilibrium potential of the CO_2_/CO acting.

**Figure 1:**
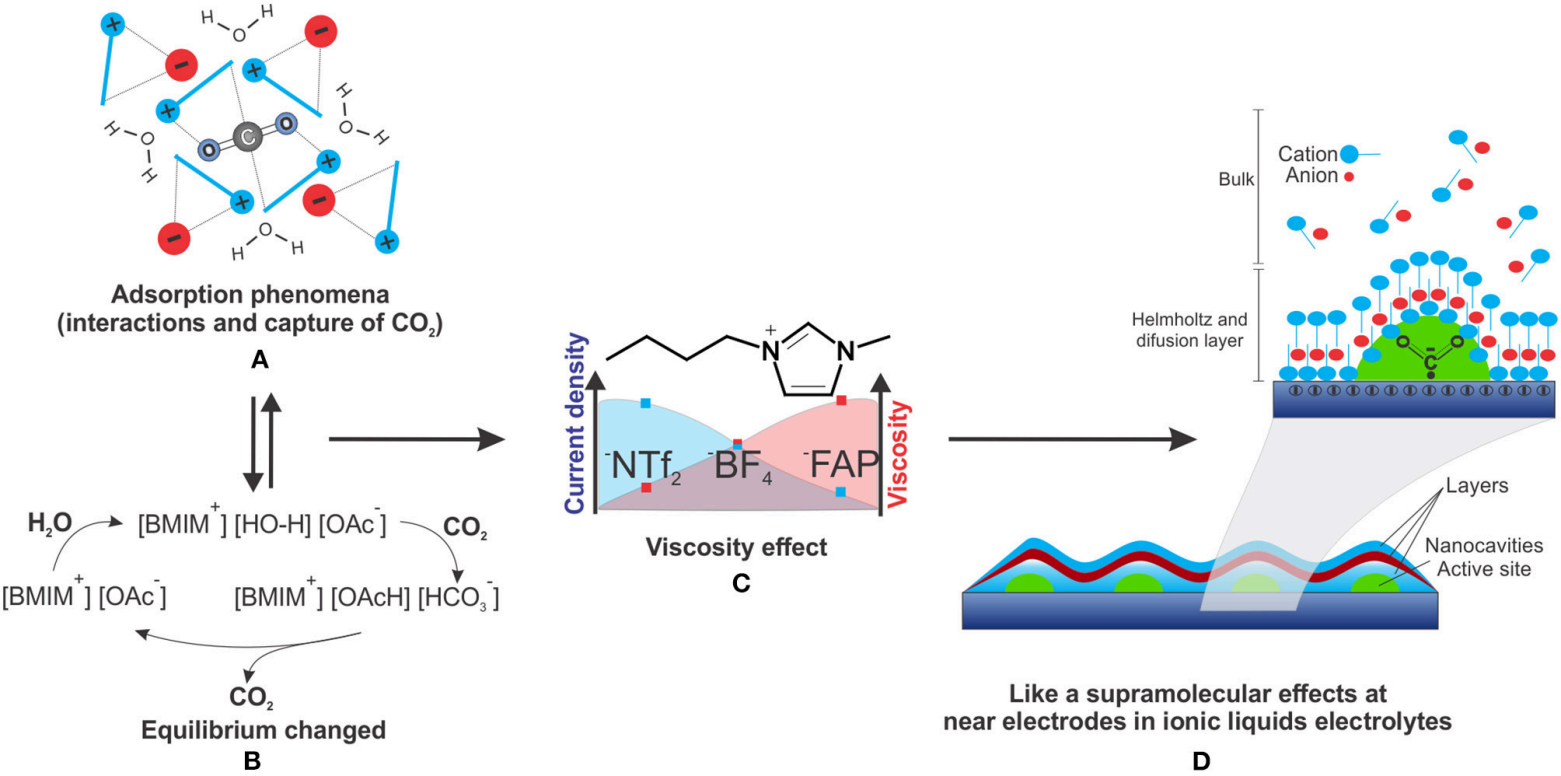
**(A)** CO_2_ adsorption phenomena in ILs, **(B)** bicarbonate equilibrium for the acetate anion in the presence of water and CO_2_, **(C)** Effect of lower viscosity and higher current density in ILs solutions and **(D)** a supramolecular-like effect near the electrode.

The lifetime of a radical is one important factor for the major efficiency in CO_2_ERR. The lifetime of ${\text{CO}}_{2}^{•-}$ was determined by pulse radiolysis time-resolved resonance Raman spectroscopy to be 10 ns (Janik and Tripathi, 2016). Furthermore, the dynamic effect of recombination depends on the surroundings (Figure 1A). A change in the surroundings is possible by an alteration of the ILs (Strehmel, 2012).

The physical absorption of CO_2_ is possible because of the ability of ILs to confine CO_2_ inside cavities near alkyl groups and aromatic protons (H4 and H5) of the IL, an interaction that does not compete with the interaction of the IL counter ion (Corvo et al., 2013).

ILs also play a role similar to surfactants near the electrode (Figure 1D), where imidazolium cations help the stabilization of CO2^•−^, avoiding the dimerization process, inhibiting oxalate production, favoring CO, and decreasing the overpotential (Sun et al., 2014) (Table 1, entry 11–12).

According to a proposed mechanism (Duong et al., 2004), ILs can chemically adsorb CO_2_ through a carboxylation process on the imidazolium C2 position for the decreases in CO_2_ERR overpotential and posterior formation of CO. Following this mechanism, other CO_2_ERR studies were made with the IL C2 position protected with a methyl group to avoid the carboxylation process (Sun et al., 2014). Higher CO formation was observed, indicating that the process does not depend exclusively on carboxylation of the C2 position.

## Basic ILs and the Bicarbonate Effect

Anion basicity is also an important issue. By adjusting this property, it is possible to obtain high adsorption values and, in some cases, a positive effect in the presence of water (Wang et al., 2011; Taylor et al., 2015). Reversible carbonate formation when using gas mixture ILs has already been proposed, considering that CO_2_ capture can form bicarbonate species in solution (Ma et al., 2011; Anderson et al., 2015). Bicarbonate species formation is more efficient and more frequent than expected when there is water contribution to the reactivity and self-organization of ILs, providing a third kind of sorption mechanism (Simon et al., 2017; Qadir et al., 2018).

The absorption parameters of CO_2_ by ILs can make efficient diffusion mass transport to the electrode surface with high adsorption possible. There are two main processes of CO_2_ adsorption by ILs: non-covalent interactions, i.e., physical adsorption of CO_2_, mainly in ILs with non-basic nucleophilic anions, such as hexafluorophosphate and bis(trifluoromethyl) sulphonyl amide (Figure 1A), and chemical adsorption by carboxylation and CO_2_ conversion to bicarbonate in proton-rich media, occurring mainly in ILs with acid protons for easy deprotonation and basic anions, such as acetate and imidazolium (Figure 1B) (Simon et al., 2017).

The role of water on CO_2_ERR has been described (Simon et al., 2017), where depending on the IL anion structure, the reaction of CO_2_ with the confined and “activated” water can shift the equilibrium to bicarbonate (Figure 1B). Water activation can even occur in some IL aqueous solutions that act as a neutral base catalyst as well as a proton buffer.

In the same study, it was also reported that basic ILs with acetate and imidazolate anions in aqueous solutions can have buffer properties. It is possible, considering that the retained water molecules by the contact ion couple are active and react reversibly with CO_2_, to form bicarbonate species in solution. Therefore, water and CO_2_ are active species in these solutions and can modify the mechanistic steps from the bicarbonate formation.

## Near Electrode Organization ILs

For desired applications, better understanding of the self-organization of ILs is crucial. Some properties, such as viscosity, conductivity, polarity, and thermic properties, are important for better understanding the ILs influences on the radical stabilization process for CO_2_ERR (Strehmel, 2012).

The diffusion of species in ILs may be strongly affected by both the macroscopic viscosity of ILs and molecular parameters related to structural phenomena, like the microviscosity (Yago and Wakasa, 2011; Strehmel, 2012). These regions play an important role when confining species near the electrode in the Helmholtz plane and diffusion layer, favoring synergistic effects capable of inducing and catalyzing specific reactions.

It was proposed that the reduction in the overpotential for CO_2_ERR when using [EMIM][BF_4_] was a result of the cation complexing with ${\text{CO}}_{2}^{•-}$ (Rosen et al., 2011). Indeed, when using 1-butyl-1-methylpyrrolodinium, a cation unable to realize π- π interactions (Tanner et al., 2016) (Table 1, entries 18–23), the overpotential decreases at comparable value than using cations able to realize it. This suggests that the interaction previously proposed by Rosen et al. (2011) is probably unlikely in the reduction of the overpotential. This leads to another assumption proposed by a different mechanism, which is an inner-sphere process (Tanner et al., 2016). This mechanism involves the previous desorption of the cation from the silver electrode surface, allowing CO_2_ to access the surface, before the irreversible CO_2_ERR. However, it is assumed in this case that ILs are free ions and not structured as ion pairs and aggregates, as usually observed in solution (Stassen et al., 2015).

When the anion of the [BMIM] IL was varied, the current density increase was observed in the following order: 1-butyl-3-methylimidazolium tris(pentafluoroethyl)trifluorophosphate ([BMIM][FAP]), [BMIM][BF_4_], and 1-butyl-3-methylimidazolium bis(trifluoromethylsulfonyl)imide ([BMIM][NTf_2_]) (Tanner et al., 2016) (Figure 1C). For the [BMIM] ILs with different anions the increase of density current (at high dilution) follows the same trend of viscosity decreasing (at low dilution) (Paduszynski and Domanska, 2014).

Such effects are also observed when varying the IL cation, with the same trend of increasing current density with the decrease in viscosity (Figure 1C) (Reche et al., 2014).

The solubility of CO_2_ in conventional ILs, such as [BMIM] and [EMIM], can increase according to the alkyl chain increase (Reche et al., 2014). The solubility is also correlated with the anion nature, increasing with the fluorination nature from the anion, indicating that CO_2_ solubility increases with charge delocalization.

The viscosity effect on lifetime, mobility, dimerization, and radical coordination in ILs was evaluated (Strehmel, 2012), where the radical lifetime and the recombination dynamic are extremely dependent on the environment. An example of this recombination is that the increase in the IL concentration caused a decrease in oxalate production from CO_2_ERR and an increase in CO (Sun et al., 2014). This indicated that the IL was able to immobilize ${\text{CO}}_{2}^{•-}$ at the electrode surface, making the dimerization process more difficult and, consequently, decreasing oxalate production.

The interaction between [IM]^+^ and ${\text{CO}}_{2}^{•-}$ was also studied (Lau et al., 2016) and the 4 and 5 positions of [IM]^+^ were able to make hydrogen bonds with the radical, providing higher current density compared to the substituted [IM]^+^ at the same positions (Table 1, entries 24–26).

The radical stabilization, increase in lifetime, mobility, and the observation that ILs of [IM]^+^ can promote hydrogen bonds with the radical lead to the idea that reactive microregions could be formed at the electrode surface.

The concept of microregions was demonstrated through theoretical calculations (Lim et al., 2018), wherein the formation of microenvironments promotes the formation of a “cage” capable of promoting CO_2_ERR. It was also demonstrated through calculations that instead of the conventional idea of an intermolecular bond between the IL and ${\text{CO}}_{2}^{•-}$, the microregion effect promotes better catalytically efficiency, even in diluted conditions. This mechanism suggests that even in high diluted solutions, there is an important relationship between the volume properties, such as resistance, solubility, gas diffusivity, and viscosity.

This corroborates the idea of a microenvironment, similar to the supramolecular structures formed in low diluted ILs. The increase in IL concentration near the electrode surface was proven by the Helmholtz and diffusion layer, which considerably increases the electrolyte concentration in this region (Figure 1D).

The idea that the electric field effect at near electrode surface leads to a local rise of the IL concentration (Lim et al., 2018), is supported by the relationship among current density, viscosity, reduction of dimerization effect, and microenvironments formation.

These increases in concentration, even in diluted solutions, are able to induce the formation of a thin organized structure on the double layer region and diffusion layer, promoting considerable local concentration increase in the solution (Yochelis et al., 2015).

The concept presented here brings a new point of view to CO_2_ERR based on concepts already known regarding the supramolecular structures of ILs, taking a step forward toward the precise determination of the CO_2_ERR mechanism in ILs based on macro and microstructuration.

## Conclusions

For CO_2_ERR, ILs play a significant role due their distinct physical chemistry properties, the tuning of the reactions conditions, the assistance with ${\text{CO}}_{2}^{•-}$ stabilization, the decrease in overpotential, and the increase in faradaic efficiency and current density.

The basicity of the anion has been shown to play an important role in CO_2_ERR, helping to obtain high adsorption values and positive effects in the presence of water. The CO_2_ capture and formation of bicarbonate species combined with the reactivity and self-organization of ILs can exhibit a different sorption process in proton-rich media, occurring mainly in ILs with acid protons and basic anions, such as acetate and imidazolate. This equilibrium with bicarbonate can be shifted with confined water activation by the IL anion structure, which acts like a neutral base catalyst as well as a proton buffer.

ILs have shown notorious participation in CO_2_ERR, being involved in distinct ways, from diluted to bare ILs. This capacity of self-organization is able to confine species and favor synergistic effects that are capable of inducing and catalyzing specific reactions. When ILs are exposed to an electric field in the case of electrocatalysis, the cited self-organization is able to form a microenvironment, even in diluted conditions, increasing the IL concentration near the electrode surface.

Further efforts are needed for understanding the global reaction mechanism, with the aim of improving the research and helping solve atmospheric CO_2_ problems, especially with regards to the generation of clean energy carriers.

## Author Contributions

All authors listed have made a substantial, direct and intellectual contribution to the work, and approved it for publication.

### Conflict of Interest Statement

The authors declare that the research was conducted in the absence of any commercial or financial relationships that could be construed as a potential conflict of interest.
